# Stress, Resilience, and Sense of Coherence in Healthcare Professionals

**DOI:** 10.3390/healthcare14101291

**Published:** 2026-05-09

**Authors:** Argyro Pachi, Christos Sikaras, Dimitra Lekka, Dimitrios Kasimis, Athanasios Tselebis, Ioannis Ilias

**Affiliations:** 1 Psychiatric Department, Sotiria General Hospital of Chest Diseases, GR-11527 Athens, Greece; irapah67@gmail.com (A.P.); lekkadim@yahoo.gr (D.L.); kasimisdimitrios@yahoo.com (D.K.); atselebis@yahoo.gr (A.T.); 2 Nursing Department, Sotiria Thoracic Diseases Hospital of Athens, GR-11527 Athens, Greece; cris.sikaras@gmail.com; 3 Department of Endocrinology, Hippokration Hospital, GR-11527 Athens, Greece

**Keywords:** stress, resilience, sense of coherence, healthcare professionals, nurses, physicians

## Abstract

Background: Among healthcare personnel, longitudinal studies have shown high levels of stress before, during and after the pandemic. The aim of this study was to examine the relationship between stress, psychological resilience, and sense of coherence in healthcare professionals. Subjects and Methods: In total, 203 healthcare professionals (63 physicians and 140 nurses) completed the Perceived Stress Scale (PSS), the Brief Resilience Scale (BRS), and the Sense of Coherence Questionnaire (SOC-13). The age, sex, and profession of the participants were recorded. Results: Women exhibited higher levels of perceived stress compared to males (18.450 ± 7.232 vs. 15.116 ± 5.662, *t*-test *p* < 0.05), as well as lower scores on the sense of coherence scale (57.525 ± 13.716 vs. 65.535 ± 12.481, *t*-test *p* < 0.05). No differences were observed with respect to profession. High levels of stress were recorded in 12.3% of workers and moderate values in 58.7%. The PSS demonstrated a negative correlation with both the SOC-13 and the BRS. The BRS showed a positive correlation with the SOC-13 (Pearson *p* < 0.01). Age showed no significant correlation. Linear regression analysis indicated that 49% of the variance in PSS was explained by SOC-13 and 3% by BRS. We subsequently investigated the hypothesis that the BRS may function as a mediator in the relationship between SOC-13 and PSS. Mediation analysis revealed that the BRS acts as a mediator in the relationship between SOC-13 and PSS. The indirect effect of BRS was statistically significant [b = −0.0770, 95% CI (−0.1312, −0.0216), *p* ≤ 0.01]. Furthermore, even in the presence of BRS mediation, the direct effect of SOC-13 on PSS remained significant [b = −0.2796, 95% CI (−0.3450, −0.2142), *p* ≤ 0.001]. Conclusions: High rates of stress, particularly in women healthcare professionals, appear to be intrinsically associated with the healthcare profession. It is likely that sense of coherence and psychological resilience can reduce stress, with resilience acting as a mediator.

## 1. Introduction

During the pandemic crisis, several studies documented, as was to be expected, high levels of stress in the general population [1,2,3] and markedly elevated levels in healthcare professionals [4,5]. Numerous studies reported significant levels of anxiety, depression, fatigue, and burnout among healthcare professionals, attributable both to the pandemic itself and to the unprecedented conditions that accompanied it [6,7,8]. In reality, high levels of psychological distress were prevalent in healthcare professionals before the pandemic, while studies conducted several months after the end of the crisis did not identify any significant improvement in their mental health [9,10,11,12]. A hypothetical, yet plausible, explanation relates to the very nature of the healthcare profession, which has been characterized as inherently stressful across time. However commonplace and accepted this explanation may be, it does not account for why the majority of healthcare workers, regardless of the intense environmental pressures they faced, continued to remain mentally healthy. The answer offered directly or indirectly by researchers was related to the protective factors or resources that healthcare professionals may develop. This answer is likely connected to two distinct theoretical approaches: the theory of salutogenesis [13,14] and the theory of positive psychology [15,16].

Positive Psychology is a relatively contemporary branch of psychological science that focuses on the study of positive emotions, individual strengths, and the conditions that promote human well-being. In contrast to traditional psychology, which was primarily centered on psychopathology and the treatment of disorders, Positive Psychology aims to understand and enhance the factors that make life meaningful and psychologically healthy [17,18]. One of the central areas of interest within Positive Psychology is psychological resilience [19,20], which “is defined as the capacity of an individual to adapt effectively and recover from adversity, stressful experiences, or traumatic events” [21,22]. Resilience is not considered an innate and immutable trait, but rather a dynamic set of skills that can be developed through experience and intervention [23,24]. Published literature has supported the existence of moderate psychological resilience among healthcare workers prior to the pandemic crisis [25]. During the pandemic, although resilience levels remained at similar levels, psychological distress increased significantly in both nurses and physicians [26,27]. Resilience functioned as a protective factor [28,29,30,31], but was insufficient on its own to offset the extreme demands placed upon healthcare workers [30].

Aaron Antonovsky defines the sense of coherence as the capacity of an individual to experience life as meaningful, comprehensible, and manageable [32,33]. This concept is regarded as a relatively stable personal characteristic [34]. In patients diagnosed with diabetes, studies “link a high sense of coherence with better management of their condition, while in patients with other chronic illnesses, a higher sense of coherence is associated with better quality of life” [35,36,37,38,39,40]. During the pandemic, lower sense of coherence levels were associated with increased psychological distress, while higher sense of coherence levels acted protectively against anxiety, depression, and psychological distress in healthcare workers [9,41,42].

Few studies have examined resilience and sense of coherence simultaneously; however, a common finding in these studies supports a positive correlation between these two protective factors for mental health [43].

In summary, the concepts of sense of coherence and resilience are central to understanding psychological adaptation in the face of adversity. Both have been extensively studied in health psychology and stress research; however, the nature of their relationship—and in particular whether resilience can mediate the relationship between sense of coherence and perceived stress—has not been thoroughly examined. Importantly, these two frameworks—salutogenesis and positive psychology—are not independent; rather, they share a common emphasis on psychological resources that enable individuals to maintain or recover wellbeing in the face of adversity. Sense of coherence provides a stable dispositional orientation toward comprehensibility, manageability, and meaningfulness that may itself foster the development of resilient responses, while resilience represents the dynamic, behavioral expression of that orientation under stress. Thus, a unified theoretical model would propose that sense of coherence acts as a distal protective resource that not only directly reduces stress appraisal, but also indirectly buffers stress by cultivating resilience capacities [43]. This conceptual integration provides the rationale for the mediation model tested in the present study.

The aim of this study was to assess levels of perceived stress in healthcare workers and to examine the relationship between resilience and sense of coherence. The hypotheses were derived from and grounded in the theoretical frameworks described above and in prior empirical evidence, as follows:

The hypotheses proposed were as follows:

**H1.**  *Sense of coherence is negatively correlated with and predicts perceived stress. Antonovsky’s salutogenic model [32,33] posits that individuals with a strong sense of coherence perceive stressors as comprehensible and manageable, thereby appraising them as less threatening. Empirical support for this hypothesis derives from studies conducted in healthcare workers during [41,42] and after [9] the pandemic.*

**H2.**  *Resilience is negatively associated with perceived stress and predicts it. Resilient individuals demonstrate greater capacity to recover from stressful experiences [21,22,24]; prior research in healthcare workers confirms that higher resilience is associated with lower perceived stress and psychological distress [28,29,30].*

**H3.**  *Resilience mediates the relationship between sense of coherence and perceived stress. Given that sense of coherence and resilience are positively correlated [43] and both independently predict lower stress, we hypothesize that sense of coherence enhances resilience, which in turn reduces perceived stress, constituting a partial mediation pathway consistent with the unified theoretical framework outlined above.*

## 2. Materials and Methods

### 2.1. Research Design

We employed a cross-sectional design with convenience sampling among medical and nursing staff at one of the largest hospitals in Greece. More in detail, the sampling approach involved sending participation invitations to all healthcare professionals at the selected institution; accordingly, the sample should be regarded as a convenience sample rather than a probability sample. The rationale for selecting the “Sotiria” Hospital was twofold: it served as the most important referral center during the pandemic period, and perceived stress levels were assessed in its personnel during the pandemic. Google Forms was used to send participation invitations to the email addresses of healthcare workers. The invitation included an anonymous link directing participants to the Google Forms research platform. The first page of the platform contained informed consent, in which the participant was required to indicate agreement to take part in the study before proceeding to complete the remaining sections. The study was conducted in the second quarter of 2025.

### 2.2. Study Participants

As part of the design of the present study, a power analysis was conducted for multiple linear regression with six independent variables, to estimate the required sample size. Based on a level of statistical significance of α = 0.05, a desired power of 1 − β = 0.80, and an effect size of f^2^ = 0.15 (per Cohen), the minimum required sample size was estimated at approximately 98 participants. Of the 250 invitations sent, 203 healthcare professionals were included in the study: 63 physicians and 140 nurses. The sample was considered adequate based on Kline’s empirical rule [44].

### 2.3. Ethical Considerations

The study complied with the following ethical principles:

– General Data Protection Regulation of the European Union (GDPR-2016/679).
– Declaration of Helsinki (revised 2008).

The study was approved by the Clinical Research Ethics Committee of the Athens Chest Diseases General Hospital “Sotiria” (Approval Number: 27463/07-10-2024).

### 2.4. Measurement Instruments

Participants first provided their sex, age, and profession, and subsequently completed the Perceived Stress Scale, the Brief Resilience Scale, and the Sense of Coherence questionnaire.

The Perceived Stress Scale (PSS) [45,46] is a widely used psychometric instrument for assessing perceived stress as experienced by the individual over the past month. The scale consists of 10 items, rated on a five-point Likert scale (0 = “never” to 4 = “very often”). The total score is derived from the sum of the individual responses, with higher scores indicating higher levels of perceived stress. Scores of 0–13 indicate low perceived stress, scores greater than 27 indicate high perceived stress, and scores of 14–26 reflect moderate stress. The Greek version of the PSS has demonstrated satisfactory reliability and validity, making it a reliable instrument for research and clinical use [47,48]. The internal consistency (Cronbach’s alpha) for the study sample was 0.880.

The Brief Resilience Scale(BRS) is a brief psychometric instrument designed for the direct assessment of psychological resilience, defined as the capacity of the individual to recover from stressful situations or difficulties [49,50]. The BRS consists of 6 items, rated on a five-point Likert scale (1 = “strongly disagree” to 5 = “strongly agree”), with three of them reverse-scored [28,49,50,51]. The total score is derived from the mean of the responses, with higher values indicating greater resilience capacity. The Greek version of the scale has demonstrated satisfactory reliability and validity [27,28,51]. For the study sample, Cronbach’s alpha was 0.881.

The Sense of Coherence Questionnaire(SOC-13) is a widely used psychometric instrument for the assessment of sense of coherence, as defined within Aaron Antonovsky’s theoretical model of salutogenesis [52]. The concept of sense of coherence refers to the degree to which an individual perceives the world as comprehensible, manageable, and meaningful. The short version of the scale comprises 13 items rated on a seven-point Likert scale. The total score is derived from the sum of the responses, with higher scores indicating a stronger sense of coherence [52,53,54]. We used the Greek version of the SOC-13, which has demonstrated satisfactory reliability and validity in other studies [53,54,55]. In the present study, Cronbach’s alpha was 0.883.

### 2.5. Statistical Analysis

Statistical analysis of the data was performed using IBM SPSS Statistics 21 (IBM SPSS Statistics for Windows, Version 21.0, Armonk, NY, USA: IBM Corp.). Initially, descriptive statistical analysis was conducted for the demographic and basic characteristics of the sample, including means, standard deviations, frequencies, and percentages. The reliability of the psychometric instruments (Perceived Stress Scale—PSS, Brief Resilience Scale—BRS, and Sense of Coherence Questionnaire—SOC-13) was assessed using Cronbach’s α internal consistency coefficient (see above). Subsequently, the normality of the distribution of continuous variables was examined using both the Kolmogorov–Smirnov test and the Shapiro–Wilk test. The Shapiro–Wilk test was selected as the primary normality criterion given its superior statistical power in samples of this size; results indicated that the distribution of the main variables did not significantly deviate from normality, thus supporting the use of parametric tests (independent-samples *t*-test, Pearson correlation, and multiple linear regression) in the analyses that followed.

To investigate the relationships among the primary variables (perceived stress, resilience, and sense of coherence), the Pearson correlation coefficient was used. In addition, multiple linear regression was performed to examine the extent to which resilience and sense of coherence predict perceived stress levels, controlling for potential confounding variables (e.g., age, sex, profession).

Furthermore, the potential mediating effect of resilience on the relationship between sense of coherence and perceived stress was examined using mediation analysis in accordance with the methodology of Andrew F. Hayes and Kristopher J. Preacher (Model 4). Specifically, the bootstrap procedure was applied with 5000 resampling iterations to estimate the indirect effects and the corresponding 95% confidence intervals. Mediation was considered statistically significant when the confidence interval did not include zero [56,57].

The level of statistical significance was set at *p* < 0.05, and all tests were two-tailed. Finally, the basic assumptions of regression were verified, including multicollinearity, independence of errors, and homoscedasticity, to ensure the validity of the results.

## 3. Results

A total of 203 healthcare professionals participated in the present study—43 men and 160 women (63 physicians and 140 nurses). Descriptive statistics are presented in Table 1. Women exhibited statistically higher values on the Perceived Stress Scale (15.116 ± 5.662 vs. 18.450 ± 7.232, *t*-test *p* < 0.01, Table 1) and lower values on the Sense of Coherence Questionnaire (65.535 ± 12.481 vs. 59.222 ± 13.829, *t*-test *p* < 0.01, Table 1). No differences were observed with respect to profession. High levels of stress were recorded in 12.3% of workers and moderate values in 58.7%.

Correlation analysis revealed statistically significant relationships among the primary variables. Specifically, perceived stress correlated negatively with resilience and sense of coherence, while a positive correlation was observed between resilience and sense of coherence (*p* < 0.01, Table 2).

A hierarchical multiple regression analysis was conducted (Table 3) to examine whether sense of coherence (SOC-13) and resilience (BRS) predict perceived stress (PSS) beyond demographic variables (sex, age, and profession).

In Step 1, the demographic variables explained a small but statistically significant proportion of the variance in perceived stress, R^2^ = 0.044. Among these variables, only sex was a significant predictor (β = 0.195, *p* = 0.007), indicating that women reported higher levels of perceived stress compared to men. Age (β = −0.080, *p* = 0.261) and profession (β = 0.034, *p* = 0.638) were not significant predictors.

In Step 2, sense of coherence and resilience were added to the model, resulting in a substantial increase in explained variance, ΔR^2^ = 0.479, with the full model explaining 52.3% of the variance in perceived stress, R^2^ = 0.523, *p* < 0.001.

In the final model, sense of coherence emerged as the strongest predictor of perceived stress (β = −0.543, *p* < 0.001), followed by resilience (β = −0.233, *p* < 0.001), indicating that higher levels of both variables are associated with lower perceived stress. Notably, after the inclusion of these psychological variables, the effects of sex (β = 0.053, *p* = 0.309), age (β = 0.016, *p* = 0.759), and profession (β = −0.050, *p* = 0.340) were no longer statistically significant.

Finally, variance inflation factor (VIF) values ranged from 1.056 to 1.902, indicating no issues with multicollinearity. The Durbin–Watson value was 2.1, indicating the absence of autocorrelation. Heteroscedasticity was examined through visual inspection of the scatter plot of standardized residuals and predicted values, and by means of the Breusch–Pagan test (*p* >0.05).

**Figure 1:**
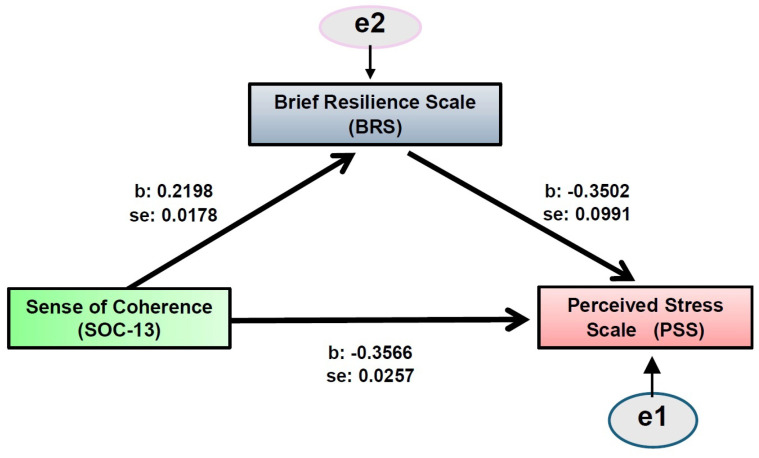
Mediation analysis of Brief Resilience Scale (BRS) on Sense of Coherence (SOC-13)—Perceived Stress Scale (PSS).

A mediation analysis (Figure 1) was conducted to examine whether resilience (BRS) mediates the relationship between sense of coherence (SOC-13) and perceived stress (PSS).

The results indicated that sense of coherence was a significant positive predictor of resilience (B = 0.2198, SE = 0.0178, t = 12.35, *p* < 0.001), suggesting that higher sense of coherence is associated with higher levels of resilience (Table 4). In addition, sense of coherence was a significant negative predictor of perceived stress (B = −0.3566, SE = 0.0257, t = −13.88, *p* < 0.001), indicating that higher SOC is associated with lower perceived stress. The direct effect of sense of coherence on perceived stress remained statistically significant after the inclusion of resilience in the model (B = −0.2796, SE = 0.0331, t = −8.43, *p* < 0.001), although it was reduced in magnitude compared to the total effect. This finding suggests partial mediation. Importantly, the indirect effect of sense of coherence on perceived stress through resilience was statistically significant (B = −0.0770, SE = 0.0274), as the 95% bootstrap confidence interval did not include zero [−0.1314, −0.0227], confirming the mediating role of resilience. The proportion of the total effect mediated by resilience was approximately 21.6%, indicating that about one-fifth of the relationship between sense of coherence and perceived stress is explained through resilience.

## 4. Discussion

The present study aimed to investigate the relationships among sense of coherence, resilience, and perceived stress among healthcare professionals, as well as to examine the mediating role of resilience in the association between sense of coherence and perceived stress. The findings of the present study revealed that 12.3% of healthcare professionals exhibited high levels of perceived stress, while the majority (58.7%) displayed moderate levels, with a mean score of 41 across the entire sample. It is noteworthy that a study in the same population at the onset of the pandemic had shown 45.5% moderate perceived stress and 4.8% high perceived stress [48]. Similarly, measurement of SOC-13 in Greek nurses during the pandemic period yielded a mean of 60.1 [58], very close to the value of 59.2 proposed by the present study. Resilience appears to display a degree of longitudinal stability, as the value of 3.36 proposed by the present study is close to values found in Greek healthcare workers during the pandemic crisis and in nurses after the end of the pandemic [28]. The findings of this study are in agreement with those of other researchers who maintain that, even following the pandemic crisis, healthcare personnel continue to experience significant psychological distress [59,60,61,62], and that administrative measures and organizational interventions are therefore necessary.

The findings of the study demonstrated that sense of coherence was a strong negative predictor of perceived stress, indicating that individuals with higher levels of coherence tend to experience lower stress. This finding is consistent with the theoretical framework of Aaron Antonovsky [32,33], according to which a strong sense of coherence enables individuals to perceive life as more comprehensible, manageable, and meaningful, thereby enhancing their capacity to cope with stressors [63,64,65]. The dominance of sense of coherence over resilience as a predictor (β = −0.543 vs. β = −0.233 in the final model) warrants theoretical consideration. Antonovsky conceptualized sense of coherence as a global, relatively stable orientation that permeates an individual’s appraisal of all life challenges, not merely specific stressful events. This dispositional breadth may explain its stronger and more consistent influence on perceived stress across diverse situations compared to resilience, which is conceptually narrower and tied more closely to specific adversity and recovery processes. Additionally, the fact that 47.7% of variance in perceived stress scale remained unexplained by the full model (R^2^ = 0.523) suggests that other factors also play important roles. These may include social support [66,67], coping style, emotional regulation strategies, sleep quality, and broader occupational and organizational factors. Future research should incorporate these variables into expanded models.

Resilience also emerged as a significant negative predictor of perceived stress, in agreement with previous research [68,69,70,71], which has supported the notion that resilient individuals are better positioned to adapt to adversity and recover from stressful experiences. Importantly, sense of coherence was found to correlate positively with resilience, indicating that individuals who perceive their environment as structured and meaningful are more likely to develop adaptive coping mechanisms. The relatively strong correlation between sense of coherence and resilience (r = 0.657, *p* < 0.001) warrants a brief discussion of construct overlap. While both constructs are protective psychological resources and share variance, they are theoretically and operationally distinct: sense of coherence is a stable dispositional orientation toward the world as comprehensible, manageable, and meaningful, whereas resilience refers to the dynamic capacity to bounce back from adversity. The high correlation may reflect the fact that a strong sense of coherence provides the cognitive and motivational foundation that supports the development of resilient responses—a relationship captured precisely by the mediation model tested in this study. The absence of multicollinearity issues (VIF values below 2) further supports their independent contributions to variance in stress.

A key finding of the study was that resilience partially mediated the relationship between sense of coherence and perceived stress. Specifically, approximately 21.6% of the total effect of sense of coherence on stress was explained through resilience. This suggests that sense of coherence not only exerts a direct protective effect against stress, but also indirectly reduces stress by enhancing resilience. Nevertheless, most of the effect remained direct, indicating that other additional mechanisms may also contribute to this relationship. One such factor could be the social support received by healthcare workers—a factor that on one hand acts protectively against stress and on the other underwent significant changes before and after the pandemic crisis, with an increase at the beginning of the pandemic through the heroization of healthcare professionals [72,73]. It should be noted that, given the cross-sectional design of the study, the mediation findings do not imply temporal precedence or causality; rather, they reflect statistically consistent associations that are in line with the proposed theoretical model. Longitudinal studies are required to confirm the causal directionality of these pathways.

It is noteworthy that demographic variables such as sex, age, and profession were not significant predictors in the final model, suggesting that psychological resources may play a more central role than demographic characteristics in explaining stress levels among healthcare professionals.

These findings have important practical implications at both individual and organizational levels. At the individual level, interventions aimed at enhancing sense of coherence and resilience may be particularly effective in reducing stress among healthcare workers [74,75,76]. For example, training programs focusing on meaning-making, coping strategies, and emotional regulation could strengthen both constructs and improve psychological well-being. At the organizational level, it is equally important to address the structural and institutional conditions that generate chronic stress. Hospital administrations can contribute by implementing policies that reduce workload demands, improve shift scheduling, promote collegial support networks, and provide access to psychological consultation services. Such measures acknowledge that stress in healthcare settings is not merely a product of individual psychological vulnerability but is shaped by the work environment itself. The integration of individual-level psychological interventions with organizational-level reforms is therefore recommended as the most comprehensive strategy for addressing healthcare professionals’ stress.

Despite its contributions, the study has certain limitations that should be acknowledged. First, the cross-sectional design does not permit the drawing of causal conclusions. In particular, while mediation analysis was employed to examine the indirect pathway from sense of coherence through resilience to perceived stress, such analysis in a cross-sectional study cannot establish temporal precedence and should be interpreted as reflecting statistical associations consistent with the proposed model rather than causal mechanisms. Longitudinal or experimental designs are required to confirm directionality. Second, the use of self-report measures may introduce response bias, including social desirability effects, particularly in the context of healthcare settings where perceived vulnerability may be underreported. Third, the study was conducted at a single tertiary care institution (“Sotiria” General Hospital of Chest Diseases, Athens), which limits the generalizability of findings to other hospitals, healthcare settings, and national contexts. Single-site data collection also introduces the possibility of institutional culture effects on the variables measured. Fourth, the convenience sampling approach—involving email invitations to hospital staff—may have introduced selection bias, as workers with higher levels of distress or disengagement may have been less likely to participate, potentially underestimating stress levels in the sample. Fifth, the cultural context of the study should be considered when interpreting the findings. The participants were Greek healthcare professionals, a population embedded in a specific cultural framework that may shape the expression and management of stress in distinctive ways. Greek culture is characterized by strong family and social bonds, which may act as protective resources against stress, while at the same time the economic and organizational pressures that have historically burdened the Greek public health system may amplify occupational stressors. The relationship between sense of coherence, resilience, and stress may therefore take on different magnitudes or forms in different cultural settings, and cross-cultural replication of these findings is warranted. Finally, the study did not include validated measures of social support, coping style, sleep quality, or organizational variables, which may account for a portion of the unexplained variance in perceived stress.

### Future Directions

The findings of this study point to several important directions for future research. First, longitudinal studies are needed to establish the temporal and causal ordering of the relationships among sense of coherence, resilience, and perceived stress. Such designs would allow researchers to determine whether interventions that enhance sense of coherence at one time point lead to subsequent increases in resilience and reductions in stress—a sequence that cannot be tested with cross-sectional data. Second, multi-site studies involving healthcare workers from different hospital types (e.g., primary care, psychiatric, oncology settings), different regions of Greece, and different countries would substantially improve the generalizability of findings and allow for cross-cultural comparisons. Third, future research would benefit from incorporating additional variables that likely contribute to the unexplained variance in perceived stress, such as social support, coping strategies, emotional regulation, sleep disturbance, job demands and resources, and organizational climate. Fourth, intervention studies testing the effectiveness of sense of coherence -enhancing and resilience-building programs—such as meaning-centered group interventions, mindfulness-based stress reduction, or cognitive-behavioral skills training—represent a high-priority area, as the present findings suggest that both constructs are modifiable targets with meaningful impact on stress outcomes. Such interventions would benefit healthcare workers themselves by reducing psychological distress, and healthcare systems more broadly by improving staff retention, quality of care, and organizational productivity. Fifth, the role of cultural and contextual factors—including the specific pressures facing the Greek public health system—warrants dedicated investigation, as these factors may moderate the relationships between psychological resources and stress outcomes in ways that have both theoretical and practical significance.

## 5. Conclusions

The present study emphasizes the critical role of sense of coherence and resilience as protective factors against stress, with resilience functioning as a significant, albeit partial, mediator in this relationship. These findings are consistent with—but, given the cross-sectional design, cannot definitively establish—a model in which sense of coherence builds resilience capacity, which in turn reduces stress appraisal. The findings suggest that perceived stress among healthcare workers may evolve into a chronic, multifactorial public health problem, with particular intensity in already overburdened healthcare systems. Importantly, the fact that demographic variables (sex, age, profession) ceased to be significant predictors once psychological resources were entered into the model underscores the potential value of targeting these resources in intervention programs. These results highlight the necessity of implementing organizational and administrative measures as well as conducting psychological intervention programs that address both individual-level psychological resources and the broader structural conditions of healthcare professionals.

## Figures and Tables

**Table 1:** Gender and Descriptive Statistics.

		Age	Perceived Stress Scale (PSS)	Brief Resilience Scale (BRS)	Sense of Coherence Questionnaire (SOC-13)
Men	Mean	38.767	15.116 *	3.500	65.535 *
	N	43	43	43	43
	Std. Deviation	11.343	5.662	0.713	12.481
Women	Mean	41.725	18.450 *	3.327	57.525 *
	N	160	160	160	160
	Std. Deviation	10.564	7.232	0.784	13.716
Total	Mean	41.099	17.744	3.364	59.222
	N	203	203	203	203
	Std. Deviation	10.773	7.050	0.771	13.829

* *T-test p < 0.01.*

**Table 2:** Correlations among Age, PSS, BRS, and SOC-13.

		Age	PSS	BRS
Perceived Stress Scale (PSS)	Pearson Correlation	−0.051		
	Sig. (2-tailed)	0.472		
	N	203		
Brief Resilience Scale (BRS)	Pearson Correlation	0.068	−0.590 **	
	Sig. (2-tailed)	0.337	<0.001	
	N	203	203	
Sense of Coherence Questionnaire (SOC-13)	Pearson Correlation	0.084	−0.699 **	0.657 **
	Sig. (2-tailed)	0.234	<0.001	<0.001
	N	203	203	203

**. *Correlation is significant at the 0.01 level (2-tailed).*

**Table 3:** Multiple Regression Analysis Predicting Perceived Stress (PSS).

Model	B	SE B	Beta	t	*p*	VIF
Step 1						
Gender (1 men vs. 2 women)	3.356	1.227	0.195	2.735	0.007	1.058
Age	−0.053	0.047	−0.080	−1.126	0.261	1.056
Profession (1 physician vs. 2 nurse)	0.520	1.105	0.034	0.471	0.638	1.099
*R^2^ = 0.044*						
Step 2						
Gender (1 men vs. 2 women)	0.913	0.896	0.053	1.019	0.309	1.120
Age	0.010	0.033	0.016	0.307	0.759	1.077
Profession (1 physician vs. 2 nurse)	−0.756	0.790	−0.050	−0.957	0.340	1.118
SOC-13	−0.277	0.035	−0.543	−7.999	<0.001	1.902
BRS	−0.355	0.100	−0.233	−3.556	<0.001	1.775
*R^2^ = 0.523 ΔR^2^ = 0.479*						

*Note. B: unstandardized coefficient; SE B: standard error; β: standardized coefficient; VIF: Variance Inflation Factor.*

**Table 4:** Mediation Analysis of the Brief Resilience Scale (BRS) on the Sense of Coherence (SOC-13)—Perceived Stress Scale (PSS) Relationship.

Variable	b	SE	t	*p*	95% CI LLCI	95% CI ULCI
SOC-13→ BRS	0.2198	0.0178	12.3539	<0.001	0.1847	0.2549
SOC-13→ PSS	−0.3566	0.0257	−13.8757	<0.001	−0.4073	−0.3059
SOC-13→ BRS → PSS	−0.3502	0.0991	−3.5350	<0.001	−0.5456	−0.1549
Effects						
Direct	−0.2796	0.0331	−8.4348	<0.001	−0.3450	−0.2142
Indirect *	−0.0770	0.0274			−0.1314	−0.0227
Total	−0.3566	0.0257	−13.8767	<0.001	−0.4073	−0.3059

* *Based on 5000 bootstrap samples.*

## Data Availability

The data are available from the corresponding author upon reasonable request due to restrictions related to patient confidentiality and institutional data protection policies.
